# Venous Thrombosis Risk after Cast Immobilization of the Lower Extremity: Derivation and Validation of a Clinical Prediction Score, L-TRiP(cast), in Three Population-Based Case–Control Studies

**DOI:** 10.1371/journal.pmed.1001899

**Published:** 2015-11-10

**Authors:** Banne Nemeth, Raymond A. van Adrichem, Astrid van Hylckama Vlieg, Paolo Bucciarelli, Ida Martinelli, Trevor Baglin, Frits R. Rosendaal, Saskia le Cessie, Suzanne C. Cannegieter

**Affiliations:** 1 Department of Clinical Epidemiology, Leiden University Medical Center, Leiden, the Netherlands; 2 Department of Orthopaedic Surgery, Leiden University Medical Center, Leiden, the Netherlands; 3 Angelo Bianchi Bonomi Hemophilia and Thrombosis Center, Fondazione IRCCS Ca’ Granda–Ospedale Maggiore Policlinico, Milan, Italy; 4 Department of Haematology, Addenbrook’s Hospital, Cambridge, United Kingdom; 5 Department of Thrombosis and Haemostasis, Leiden University Medical Center, Leiden, the Netherlands; 6 Department of Medical Statistics and Bioinformatics, Leiden University Medical Center, Leiden, the Netherlands; University of Glasgow, UNITED KINGDOM

## Abstract

**Background:**

Guidelines and clinical practice vary considerably with respect to thrombosis prophylaxis during plaster cast immobilization of the lower extremity. Identifying patients at high risk for the development of venous thromboembolism (VTE) would provide a basis for considering individual thromboprophylaxis use and planning treatment studies.

The aims of this study were (1) to investigate the predictive value of genetic and environmental risk factors, levels of coagulation factors, and other biomarkers for the occurrence of VTE after cast immobilization of the lower extremity and (2) to develop a clinical prediction tool for the prediction of VTE in plaster cast patients.

**Methods and Findings:**

We used data from a large population-based case–control study (MEGA study, 4,446 cases with VTE, 6,118 controls without) designed to identify risk factors for a first VTE. Cases were recruited from six anticoagulation clinics in the Netherlands between 1999 and 2004; controls were their partners or individuals identified via random digit dialing. Identification of predictor variables to be included in the model was based on reported associations in the literature or on a relative risk (odds ratio) > 1.2 and *p* ≤ 0.25 in the univariate analysis of all participants. Using multivariate logistic regression, a full prediction model was created. In addition to the full model (all variables), a restricted model (minimum number of predictors with a maximum predictive value) and a clinical model (environmental risk factors only, no blood draw or assays required) were created. To determine the discriminatory power in patients with cast immobilization (*n =* 230), the area under the curve (AUC) was calculated by means of a receiver operating characteristic. Validation was performed in two other case–control studies of the etiology of VTE: (1) the THE-VTE study, a two-center, population-based case–control study (conducted in Leiden, the Netherlands, and Cambridge, United Kingdom) with 784 cases and 523 controls included between March 2003 and December 2008 and (2) the Milan study, a population-based case–control study with 2,117 cases and 2,088 controls selected between December 1993 and December 2010 at the Thrombosis Center, Fondazione IRCCS Ca’ Granda–Ospedale Maggiore Policlinico, Milan, Italy.

The full model consisted of 32 predictors, including three genetic factors and six biomarkers. For this model, an AUC of 0.85 (95% CI 0.77–0.92) was found in individuals with plaster cast immobilization of the lower extremity. The AUC for the restricted model (containing 11 predictors, including two genetic factors and one biomarker) was 0.84 (95% CI 0.77–0.92). The clinical model (consisting of 14 environmental predictors) resulted in an AUC of 0.77 (95% CI 0.66–0.87). The clinical model was converted into a risk score, the L-TRiP(cast) score (Leiden–Thrombosis Risk Prediction for patients with cast immobilization score), which showed an AUC of 0.76 (95% CI 0.66–0.86). Validation in the THE-VTE study data resulted in an AUC of 0.77 (95% CI 0.58–0.96) for the L-TRiP(cast) score. Validation in the Milan study resulted in an AUC of 0.93 (95% CI 0.86–1.00) for the full model, an AUC of 0.92 (95% CI 0.76–0.87) for the restricted model, and an AUC of 0.96 (95% CI 0.92–0.99) for the clinical model. The L-TRiP(cast) score resulted in an AUC of 0.95 (95% CI 0.91–0.99).

Major limitations of this study were that information on thromboprophylaxis was not available for patients who had plaster cast immobilization of the lower extremity and that blood was drawn 3 mo after the thrombotic event.

**Conclusions:**

These results show that information on environmental risk factors, coagulation factors, and genetic determinants in patients with plaster casts leads to high accuracy in the prediction of VTE risk. In daily practice, the clinical model may be the preferred model as its factors are most easy to determine, while the model still has good predictive performance. These results may provide guidance for thromboprophylaxis and form the basis for a management study.

## Abstract 2

Using three population-based case-control studies, Banne Nemeth and colleagues derive and validate a clinical prediction score (L-TRiP(cast)) for venous thrombosis risk.

## Editors' Summary

**Background:**

Blood normally flows smoothly around the human body, but when a cut or other injury occurs, proteins called clotting factors make the blood gel (coagulate) at the injury site. The resultant clot (thrombus) plugs the wound and prevents blood loss. Sometimes, however, a thrombus forms inside an uninjured blood vessel and partly or completely blocks the blood flow. Clot formation inside one of the veins deep in the body (usually in a leg) is called deep vein thrombosis (DVT). DVT, which can cause pain, swelling, and redness in the affected limb, is treated with anticoagulants, drugs that stop the clot growing. If left untreated, part of the clot can break off and travel to the lungs, where it can cause a life-threatening pulmonary embolism. DVT and pulmonary embolism are known collectively as venous thromboembolism (VTE). Risk factors for VTE include age, oral contraceptive use, having an inherited blood clotting disorder, and prolonged inactivity (for example, being bedridden). An individual’s lifetime risk of developing VTE is about 11%; 10%–30% of people die within 28 days of diagnosis of VTE.

**Why Was This Study Done?:**

Clinicians cannot currently accurately predict who will develop VTE, but it would be very helpful to be able to identify individuals at high risk for VTE because the condition can be prevented by giving anticoagulants before a clot forms (thromboprophylaxis). The ability to predict VTE would be particularly useful in patients who have had a lower limb immobilized in a cast after, for example, breaking a bone. These patients have an increased risk of VTE compared to patients without cast immobilization. However, their absolute risk of VTE is not high enough to justify giving everyone with a leg cast thromboprophylaxis because this therapy increases the risk of major bleeds. Here, the researchers investigate the predictive value of genetic and environmental factors and levels of coagulation factors and other biomarkers on VTE occurrence after cast immobilization of the lower leg and develop a clinical tool for the prediction of VTE in patients with plaster casts.

**What Did the Researchers Do and Find?:**

The researchers used data from the MEGA study, a study of risk factors for VTE, to build a prediction model for a first VTE in patients with a leg cast; the prediction model included 32 predictors (the full model). They also built a restricted model, which included only 11 predictors but had maximum predictive value, and a clinical model, which included 14 environmental predictors that can all be determined without drawing blood or undertaking any assays. They then determined the ability of each model to distinguish between patients with a leg cast who did and did not develop VTE using receiver operating characteristic (ROC) curve analysis. The area under the curve (AUC) for the full model was 0.85, for the restricted model it was 0.85, and for the clinical model it was 0.77. (A predictive test that discriminates perfectly between individuals who do and do not subsequently develop a specific condition has an AUC of 1.00; a test that is no better at predicting outcomes than flipping a coin has an AUC of 0.5.) Similar or higher AUCs were obtained for all the models using data collected in two independent studies. Finally, the researchers converted the clinical model into a risk score by giving each variable in the model a numerical score. The sum of these scores was used to stratify individuals into categories of low or high risk for VTE. With a cutoff of 9 points, the risk score correctly identified 80.8% of the patients in the MEGA study with a plaster cast who developed VTE and 60.8% of the patients who did not develop VTE.

**What Do These Findings Mean?:**

Some aspects of this study may limit the accuracy of its findings. For example, no information was available about which patients with a plaster cast received thromboprophylaxis. Nevertheless, these findings suggest that information on environmental risk factors, coagulation factors, and genetic determinants can be used to predict VTE risk in patients with a leg cast with high accuracy. Importantly, the risk score derived and validated by the researchers, which includes only predictors that can be easily determined in clinical practice, may help clinicians decide which patients with a leg cast should receive thromboprophylaxis and which should not be exposed to the risk of anticoagulant therapy, until an unambiguous guideline for these patients becomes available.

**Additional Information:**

This list of resources contains links that can be accessed when viewing the PDF on a device or via the online version of the article at http://dx.doi.org/10.1371/journal.pmed.1001899.

- The US National Heart, Lung, and Blood Institute provides information on deep vein thrombosis (including an animation about how DVT causes pulmonary embolisms) and on pulmonary embolism
- The UK National Health Service Choices website has information on deep vein thrombosis (including personal stories) and on pulmonary embolism
- The US non-profit organization National Blood Clot Alliance provides detailed information about deep vein thrombosis and pulmonary embolism for patients and professionals and includes a selection of personal stories about these conditions
- MedlinePlus has links to further information about deep vein thrombosis and pulmonary embolism (in English and Spanish)
- Wikipedia has a page on ROC curve analysis (note that Wikipedia is a free online encyclopedia that anyone can edit; available in several languages)
- More information about the MEGA study is available

## Introduction

The incidence of venous thromboembolism (VTE) is estimated to be 1–2 per 1,000 person-years and increases with age up to 1% per year in the elderly [1]. An individual’s lifetime risk for the development of VTE is about 11% [1–3]. Multiple genetic and environmental risk factors, including cast immobilization, have been identified in etiologic research. However, the presence of one risk factor is generally not sufficient for the development of a thrombotic event. Only when multiple risk factors have accumulated, some of which may interact in a synergistic way, and the “thrombotic threshold” is crossed will thrombosis occur [1]. Although we understand this mechanism in general, we cannot accurately predict which individuals will develop VTE [3]. Such knowledge would be of use, as it allows targeted thrombosis prevention.

Recently, Hippisley-Cox and Coupland developed a risk prediction algorithm to estimate future risk of VTE in the general population. This prediction model included 15 environmental risk factors and resulted in a receiver operating characteristic (ROC) area under the curve (AUC) statistic of 0.75 [4]. Earlier, the Padua prediction score included similar risk factors in a risk assessment model for VTE in hospitalized medical patients [5]. In addition to these prediction models, which included only environmental predictors, there have been a few studies that investigated the added value of biomarkers. Recently, de Haan et al. developed a risk model that incorporated thrombosis-associated single nucleotide polymorphisms (SNPs) combined with environmental risk factors, which reached an AUC statistic of 0.82 in the general population [6]. The role of factor VIII, D-dimer, prothrombin fragment 1 + 2, platelet count, and hemoglobin level in predicting VTE has mainly been studied in patients with cancer [7–9].

Using a prediction model for first VTE in the general population is not efficient considering the heterogeneity of the condition and the rarity of disease in the general population. However, in more homogeneous high risk groups, such as patients with cast immobilization, prediction of VTE can be useful and cost-effective. Our recent study showed an 8-fold increased risk of VTE in patients with below-knee cast immobilization [10]. In terms of absolute risk, VTE incidence rates reported in these patients vary strongly depending on study design and definition of the event (asymptomatic or symptomatic). A recent meta-analysis reported a rate of symptomatic VTE during cast immobilization that varied between 0% and 5.5% [11]. The risk of VTE during cast immobilization is probably not large enough to justify anticoagulant prophylaxis in all patients with plaster cast, as the bleeding risk will also be considerable (0.3% major bleeding) [12,13]. Therefore, it would be beneficial to identify those at high risk and to offer targeted, individualized therapy.

The purpose of this study was to investigate the predictive value of genetic and environmental risk factors, coagulation factors, and other biomarkers for the development of VTE after cast immobilization of the lower extremity. We developed several models: in addition to a full model, we also created a restricted model in which we tried to find the optimal balance between maximum predictive value and a minimum number of (all types of) predictor variables and a clinical model that contained only predictors that are easy to determine in clinical practice. Finally, we validated the models in two independent datasets.

## Methods

### Study Design

For developing the model, data from a large population-based case–control study, the MEGA study (Multiple Environmental and Genetic Assessment of risk factors for venous thrombosis) were used (S1 Analysis Plan). Details of this study have been published previously [14–16]. In short, 4,956 consecutive patients aged 18 to 70 y with a first deep vein thrombosis (DVT), pulmonary embolism (PE), or both were recruited from six anticoagulation clinics in the Netherlands between 1 March 1999 and 31 August 2004. The diagnosis of DVT or PE was confirmed by (Doppler) ultrasonography, ventilation/perfusion scan, angiography, or spiral CT scan. The control group (*n =* 6,297) consisted of partners of participating patients and other controls who were identified using a random digit dialing method; controls were frequency matched to cases with respect to sex and age. Approval for this study was obtained from the Medical Ethics Committee of the Leiden University Medical Center, and all participants provided written informed consent.

### Data Collection and Laboratory Analysis

All participants completed a questionnaire on risk factors for VTE that included questions on (potential) risk factors such as trauma, immobilization (including plaster cast and location), (orthopedic) surgery, current use of (any) medication, and comorbidity in the past year before the venous thrombotic event.

In patients and controls included from the start of the study until May 31, 2002, a blood sample was collected approximately 3 mo after discontinuation of oral anticoagulant therapy. In patients who were still on anticoagulant therapy 1 y after the event, blood was drawn during treatment. Detailed information on laboratory analyses of coagulation factors and hemorheologic and other markers can be found in S1 Laboratory Analyses. For patients and controls included after June 1, 2002, and for patients who were unable to visit the clinic, DNA was collected by means of buccal swabs sent by mail. The factor V Leiden (F5, rs6025) and prothrombin G20210A (F2, rs1799963) mutations were measured simultaneously by a multiplex polymerase chain reaction using the TaqMan assay [17]. ABO blood type was also analyzed using the TaqMan assay [18].

### Model Derivation

#### Development of the full prediction model

All prediction models were developed using the whole MEGA study population, with the exclusion of 689 individuals with multi-trauma, plaster cast of the arm or back, plaster cast *after* the occurrence of thrombosis, or use of anticoagulation medication during blood collection. In total, 4,446 cases and 6,118 controls were included in the analysis. Multiple imputation techniques were used for missing values. In the imputation step, skewed variables were transformed (five datasets were imputed, and results were pooled according to Rubin’s rules) [19].

Because the subset of individuals with plaster cast was small (*n =* 230), we were not able to test our model without imputed data in this specific group. Too many patients were missing one or more variables, and logistic regression analyses were not possible. However, results were consistent in the entire MEGA study population with and without the imputed data. Moreover, we checked all imputed data for errors. Univariate regression for all predictors was similar in the entire MEGA population when we performed regression analyses with and without imputed data. Detailed information on missing data can be found in S1 Data.

Controls were frequency matched on age and sex, meaning that the age and sex distribution of the control group was similar to that of the patient group. The age and sex distribution of the control group was therefore different from that of the general population (e.g., relatively older age and more females). In order to use age and sex as predictor variables, we needed a control group in which the age and sex distribution reflected the general population. For this we weighted the control individuals (for age and sex) to the age and sex distribution of the Dutch population in 2001 (Statistics Netherlands). Weights were calculated by dividing the proportion of individuals in a certain age- and sex-specific stratum in the Dutch population by the stratum-specific proportion of individuals in the MEGA study control group. For example, in the Dutch population, 1.2% of all inhabitants aged 18 and 70 y (same age range as our study) were 30-y-old males. In the MEGA study, this proportion was 0.8%, giving these individuals in our study a weight of 1.5 (1.2% divided by 0.8%). This approach is called direct standardization. Using this approach, younger control individuals were assigned a weight above one, and older control individuals were assigned a weight below one (stratum-specific weights can be found in S1 Weights). This way we corrected for the “oversampling” of older control individuals (due to frequency matching) and created a control group with the same age and sex distribution as that of the Dutch population in 2001. We subsequently performed weighted logistic regression analyses incorporating age and sex as predictor variables in our prediction model.

#### Derivation process

**Fig 1:**
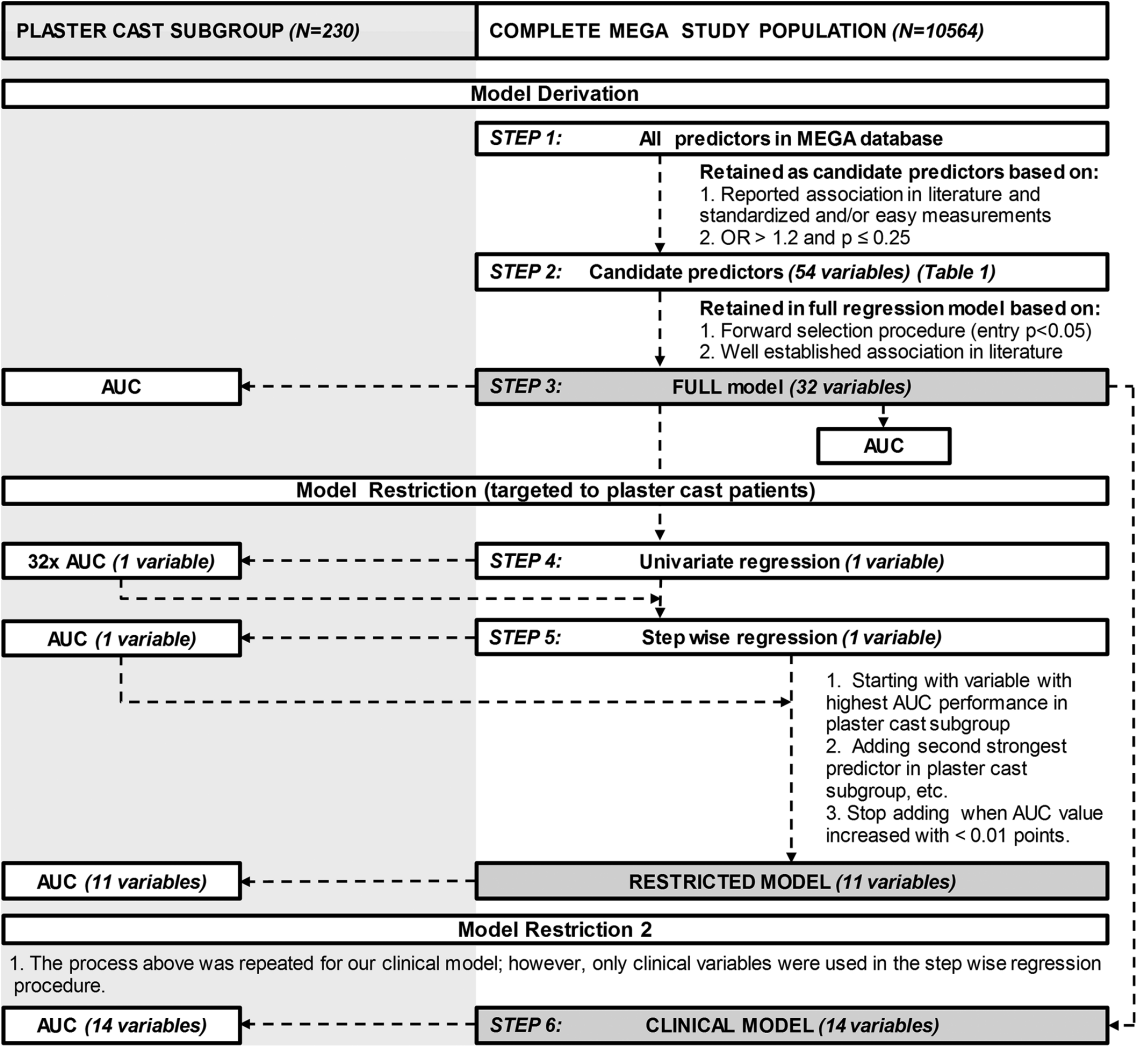
Flowchart of the prediction model derivation process.

For the development of the derivation models, the whole MEGA study population was used rather than the plaster cast subgroup, to avoid overfitting in the derivation process. Fig 1 shows a flowchart of the model derivation process. Identification of candidate predictor variables (see Table 1) was based on (1) reported associations with the occurrence of VTE in the literature and standardized and easy measurement or (2) finding an odds ratio (OR) > 1.2 (highest versus lowest category) and a *p*-value ≤ 0.25 between cases and controls in the overall MEGA study population using weighted logistic regression (Fig 1, step 1). Continuous predictors such as age and body mass index (BMI) were categorized, biomarker values were split into tertiles based on control individuals, and protein S and protein C antigen levels were dichotomized (< 65 versus ≥65 IU/dl). The variable “plaster cast” was classified as no plaster cast, complete leg cast, lower leg cast, circular knee cast, or foot cast, resulting in discrimination between different locations (more/less immobilization). Related clinical factors with a similar OR in the multivariate model were combined into one variable. The variables rheumatoid arthritis, chronic kidney disease, chronic obstructive pulmonary disease (COPD), and multiple sclerosis were combined into the variable “comorbidity”; previous heart attack and angina pectoris into “cardiovascular disease”; stroke and transient ischemic attack (TIA) into “cerebrovascular events”; and urinary tract infection/cystitis, pyelonephritis, arthritis, bursitis, inflammation of other body parts, and tropical diseases into “inflammatory disease.”

**Table 1:** Candidate predictor variables

Category	Candidate Predictor Variable
**Environmental predictor variables**	
	Age
	Sex
	Smoking
	Varicose veins
	Cancer within the past 5 y
	Congestive heart failure
	Comorbidity (rheumatoid arthritis, chronic kidney disease, COPD, multiple sclerosis)
	Cardiovascular disease (heart attack and angina pectoris)
	Cerebrovascular events (stroke and TIA)
	BMI
	Claudication
	Family history of VTE (first-degree relative)
	Hospital admission within the past 3 mo
	Bedridden within the past 3 mo
	Paralysis (partial)
	Surgery within the past 3 mo
	Current pregnancy or puerperium
	Current use of antipsychotic medication
	Current use of tamoxifen
	Current use of hormonal replacement therapy
	Current use of oral contraceptives
	Superficial vein thrombosis
	Plaster cast and location (no plaster cast, complete leg cast, lower leg cast, circular knee cast, or foot cast)
	Hepatitis
	Pneumonia
	Inflammatory disease (urinary tract infection/cystitis, pyelonephritis, arthritis, bursitis, inflammation of other body parts, and tropical diseases)
**Hemorheologic and coagulation predictor variables**	
	Fibrinogen activity
	Factor VIII activity and antigen level
	Von Willebrand factor antigen level
	Factor IX antigen mean
	Protein S antigen mean
	Factor II activity
	Factor VII activity
	Factor X antigen level
	Protein C activity
	Factor XI activity
	Hematocrit
	White blood cell count
	Percentage/number lymphocytes
	Percentage/number monocytes
	Percentage/number granulocytes
	Red blood cell count
	Hemoglobin level
	Mean cell volume
	Mean cell hemoglobin
	Mean cell hemoglobin concentration
	Red cell distribution width
	Antithrombin activity
	Total homocysteine
	Total cysteine
	Methionine
**Genetic predictor variables**	
	Factor V Leiden mutation
	Prothrombin mutation
	Non-O blood type

The full prediction model was created using a forward selection procedure (entry *p <* 0.05) with the candidate biomarkers and genetic and clinical variables. Of all the variables that were not included in the model by this forward selection, some predictors were nevertheless retained in the full model because of a well-established reported association with the occurrence of VTE in the literature (Fig 1, step 2).

#### Calculating the discriminative value

To determine the magnitude of discrimination of this model, an AUC (c-statistic) was calculated by means of a ROC, based on the predictions from the multiple logistic regression models. ROC curves were created both in the entire study population and in the plaster cast subgroup only, for which regression coefficients of the model developed in the total MEGA study population were used (Fig 1, step 3).

### Model Restriction

#### Models targeted to plaster cast patients: clinical and restricted models

From this full model, we developed two reduced sub-models specially targeted to plaster cast patients, i.e., the restricted model and the clinical model. For the development of the restricted model, we used as candidate variables the 32 variables included in our full model (including biomarkers and genetic variables). We performed a forward selection procedure. Models were fitted using all MEGA study individuals, but variables were selected based on the increase in AUC in the plaster cast subset of patients. This means that we started by fitting all 32 variables separately with a univariate logistic regression analysis using all MEGA study individuals. For each of the 32 predictors, we calculated the AUC in the subgroup of plaster cast patients (Fig 1, step 4). The variable corresponding to the highest AUC was then selected in the model (Fig 1, step 5). This procedure was repeated by subsequently adding the next strongest predictor until the AUC value in the plaster cast population increased by less than 0.01 points. Age and sex were forced (at first) in the model because of clinical importance. Variables were also selected based on their availability in our validation cohorts. For instance, when two variables performed the same in our plaster cast subgroup in the MEGA study, we chose to select the predictor that was also available in our validation cohorts. The model obtained in this way is the restricted model.

The clinical model was developed in the same way as the restricted model with the exception that only environmental predictor variables from the full model were used. Biomarkers and genetic variables were not included (Fig 1, step 6).

In this way we were able to develop models targeted to the plaster cast subpopulation, while the regression coefficients were stable because they were derived from the entire MEGA population [20].

#### Clinical risk score for plaster cast patients: the L-TRiP(cast) score

Additionally, we developed a risk score, the L-TRiP(cast) score (Leiden–Thrombosis Risk Prediction for patients with cast immobilization score), in which risk points are based on the regression coefficients (betas) for predictor variables in the clinical multivariate logistic model. We used the following scoring: 0.20 < beta ≤ 0.75, 1 point; 0.75 < beta ≤ 1.25, 2 points; 1.25 < beta ≤ 1.75, 3 points; 1.75 < beta ≤ 2.25, 4 points; beta > 2.25, 5 points. The L-TRiP(cast) score was the sum of these points across the predictor variables. The sensitivity, specificity, positive and negative predictive values, and positive and negative likelihood ratios were calculated for different cutoff points of the L-TRiP(cast) score assuming an incidence of 2.5% for VTE in plaster cast patients, which is the reported incidence from a Cochrane meta-analysis [13].

#### Model validation

Validation was performed in two other case–control studies of the etiology of VTE: the THE-VTE study [21,22] and the Milan study [23] (both published in detail previously). The THE-VTE study is a two-center, population-based case–control study that was performed in Leiden, the Netherlands, and Cambridge, United Kingdom. Valid information on all environmental risk factors was available for all 784 cases and 523 controls who were enrolled in the study between March 2003 and December 2008. The Milan study is also a population-based case–control study: 2,117 cases and 2,088 controls were enrolled between December 1993 and December 2010 at the Thrombosis Center, Fondazione IRCCS Ca’ Granda–Ospedale Maggiore Policlinico, Milan, Italy. In addition to information on environmental risk factors, data on biomarkers and genetic predictors were collected in this study. In the Milan study, all genetic predictors and factor VIII activity were measured, and most environmental risk variables were known. Only Von Willebrand factor antigen level, red cell distribution width, percentage of monocytes, factor XI activity, and total cysteine were not available. In the Milan study, the following variables were not recorded: cancer within the past 5 y, comorbidity, cerebrovascular events, hospital admission within the past 3 mo, paralysis, pregnancy, superficial vein thrombosis, hepatitis, and pneumonia. The variable smoking was coded as yes/no, family history of VTE was coded as yes/no, and information on type of plaster cast of the lower extremity (i.e., complete versus lower leg) was not available. For each individual, the different prognostic scores were calculated using the regression coefficients derived in the MEGA study.

Analyses were performed in IBM SPSS Statistics for Windows, version 20.0. The weighted analyses were performed in Stata, version 12.

## Results

### Study Population

In the model derivation analysis, 4,446 cases and 6,118 controls were included. Of the cases, 2,606 (58.6%) were diagnosed with DVT, 1,452 (32.7%) had PE, and 388 (8.7%) had both. Plaster cast immobilization of the lower extremity was present in 194 patients and 36 control individuals, mainly due to traumatic events. Among these patients, 131 (67%) individuals developed DVT, 44 (23%) PE, and 19 (10%) both. The predictors that had the highest prevalence among cases were smoking, presence of varicose veins, being overweight, family history of thrombosis (first-degree relative), use of oral contraceptives, cancer in the past 5 y, and comorbidity. Frequencies of these variables in controls were much lower. Further baseline characteristics, including coagulation markers and genetic predictor variables, can be found in S1 Table.

### Model Derivation

In univariate analyses, all 54 candidate predictor variables were significantly (*p <* 0.25) associated with the occurrence of VTE, with the exception of protein S antigen, percentage/number of lymphocytes and granulocytes, hemoglobin level, total homocysteine and antithrombin activity.

Out of these candidate predictors, 32 variables were retained in our full prediction model; these variables are listed in Table 2. The predictors cerebrovascular events, congestive heart failure, hepatitis, current use of tamoxifen, and non-O blood type were not significantly associated with VTE. Nevertheless, these were retained in the model because of a clear association with VTE in the literature. Factors most strongly associated with VTE, e.g., with the highest relative risk in this full model, were cancer within the past 5 y (OR 4.8, 95% CI 3.6–6.5), hospital admission within the past 3 mo (OR 3.6, 95% CI 2.7–4.7), current use of oral contraceptives (OR 7.3, 95% CI 6.0–8.8), pregnancy or puerperium (OR 6.1, 95% CI 4.0–9.5), complete leg plaster cast (OR 11.1, 95% CI 4.0–30.8), and factor V Leiden mutation (OR 5.7, 95% CI 1.6–19.7). S2 Table shows the univariate and multivariate ORs for the full logistic regression model in the MEGA study population. The predictive value of the full regression model resulted in an AUC of 0.85 (95% CI 0.77–0.92) in plaster cast patients and 0.88 (95% CI 0.87–0.89) in the entire MEGA population (Table 3).

**Table 2:** Overview of predictor variables in each model.

Category	Predictor Variable	Model		
		Full	Restricted	Clinical
**Environmental predictor variables**				
	Age	**×**	**×**	**×**
	Sex	**×**	**×**	**×**
	BMI	**×**	**×**	**×**
	Smoking	**×**	** **	** **
	Varicose veins	**×**	** **	** **
	Cancer within the past 5 y	**×**	** **	**×**
	Congestive heart failure	**×**	** **	** **
	Comorbidity (rheumatoid arthritis, chronic kidney disease, COPD, multiple sclerosis)	**×**	** **	**×**
	Cerebrovascular events (stroke and TIA)	**×**	** **	** **
	Family history of VTE (first-degree relative)	**×**	**×**	**×**
	Hospital admission within the past 3 mo	**×**	** **	**×**
	Bedridden within the past 3 mo	**×**	**×**	**×**
	Paralysis (partial)	**×**	** **	** **
	Surgery within the past 3 mo	**×**	**×**	**×**
	Pregnancy or puerperium	**×**	** **	**×**
	Current use of antipsychotic medication	**×**	** **	** **
	Current use of tamoxifen	**×**	** **	** **
	Current use of hormonal replacement therapy	**×**	** **	** **
	Current use of oral contraceptives	**×**	**×**	**×**
	Superficial vein thrombosis	**×**	** **	**×**
	Hepatitis	**×**	** **	** **
	Pneumonia	**×**	** **	**×**
	Plaster cast and location (no plaster cast, complete leg cast, lower leg cast, circular knee cast, or foot cast)	**×**	**×**	**×**
**Hemorheologic and coagulation predictor variables**				
	Factor VIII activity	**×**	**×**	** **
	Von Willebrand factor antigen level	**×**	** **	** **
	Factor XI activity	**×**	** **	** **
	Percentage of monocytes	**×**	** **	** **
	Total cysteine	**×**	** **	** **
	Red cell distribution width	**×**	** **	** **
**Genetic predictor variables**				
	Factor V Leiden mutation	**×**		** **
	Prothrombin mutation	**×**	**×**	** **
	Non-O blood type	**×**	**×**	** **

**Table 3:** AUC values of the full, restricted, and clinical models, both in all individuals and in the plaster cast subgroup.

Model	All Individuals		Plaster Cast Subgroup	
	AUC	95% CI	AUC	95% CI
Full model	0.88	0.87–0.89	0.85	0.77–0.92
Restricted model			0.84	0.77–0.92
Clinical model			0.77	0.66–0.87
L-TRiP(cast) score			0.76	0.66–0.86

### Restricted and Clinical Models

**Fig 2:**
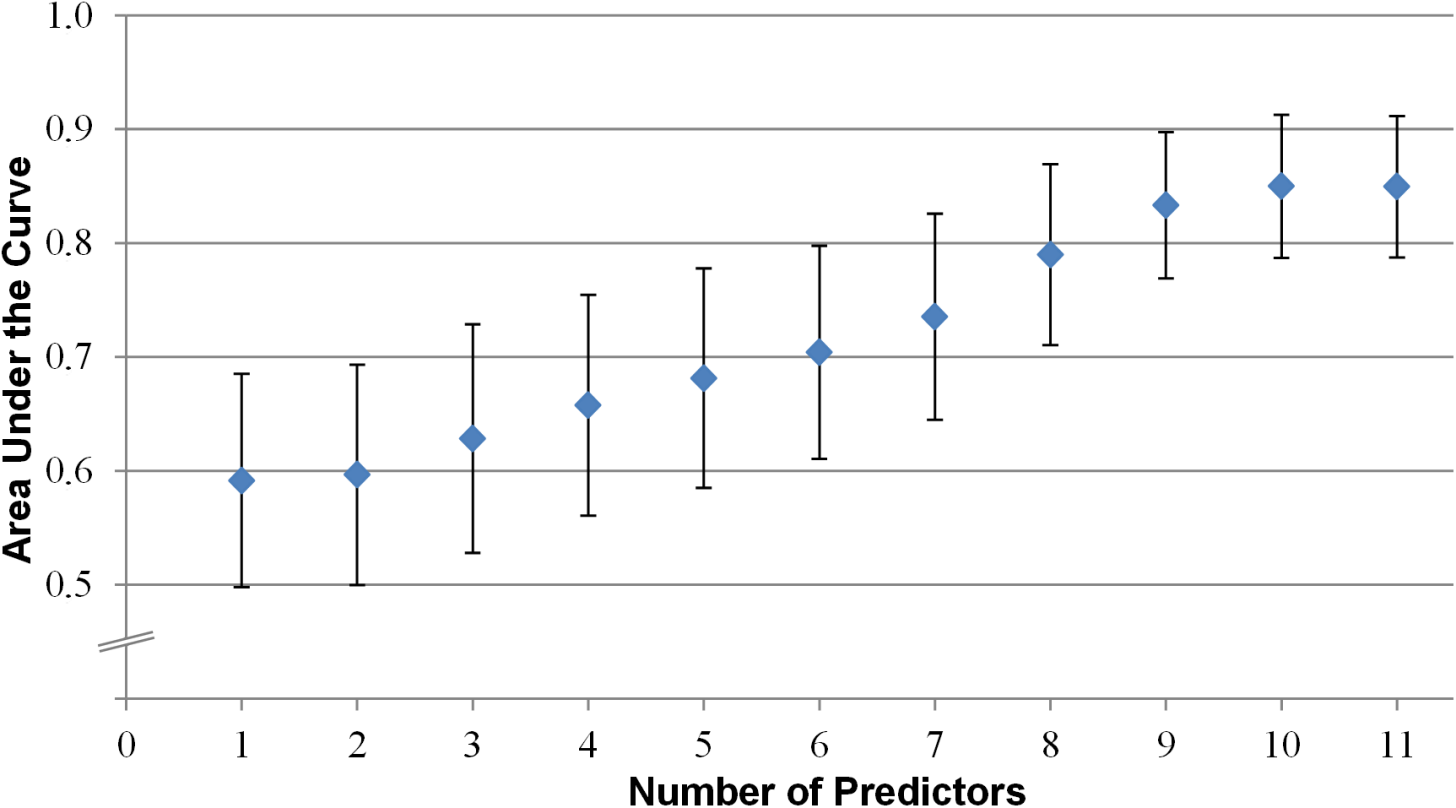
AUC value after addition of each predictor into the restricted model. Vertical bars represent 95% CIs. Predictors: (1) age, (2) sex, (3) plaster cast and location, (4) prothrombin mutation, (5) current use of oral contraceptives, (6) family history of VTE (first-degree relative), (7) factor VIII activity, (8) bedridden within the past 3 mo, (9) surgery within the past 3 mo, (10) non-O blood type, (11) BMI.

The AUC of our restricted model in plaster cast patients reached a maximum of 0.84 (95% CI 0.77–0.92) (Table 3). The restricted model comprised 11 predictor variables: age, sex, plaster cast and location, BMI, non-O blood type, current use of oral contraceptives, factor VIII activity, surgery within the past 3 mo, prothrombin mutation, family history of VTE (first-degree relative), and bedridden within the past 3 mo (see Table 2). Fig 2 shows the AUC value after each addition of a predictor into the restricted model. The clinical model consisted of 14 environmental predictor variables (see Table 2). In plaster cast patients, this model reached an AUC of 0.77 (95% CI 0.66–0.87) (Table 3).

### L-TRiP(cast) Score

**Fig 3:**
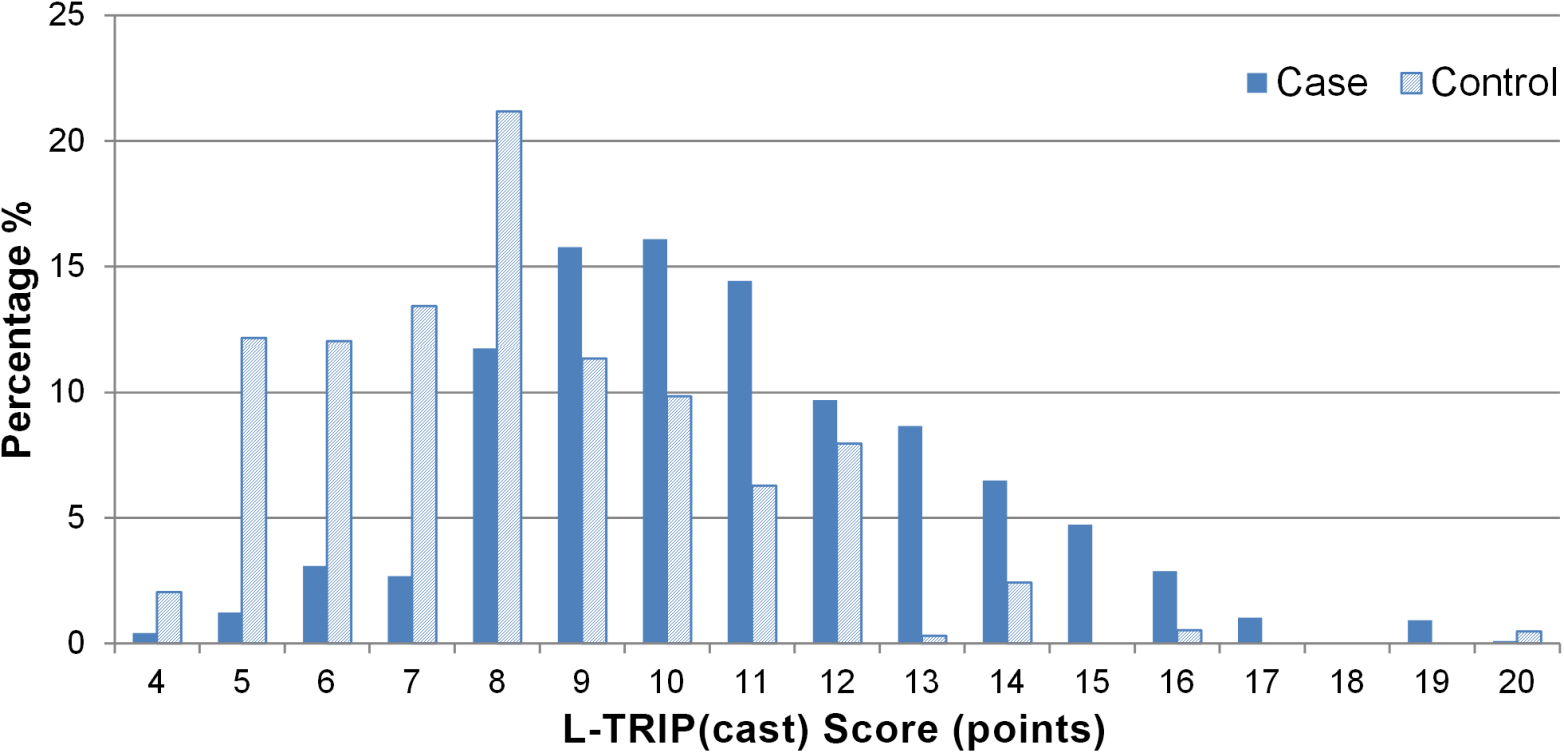
Distribution of individual L-TRiP(cast) scores in the plaster cast subgroup derived from the MEGA study.

Based on the regression coefficients in the clinical logistic regression model, the L-TRiP(cast) score was developed (Table 4). For instance, a 40-y-old male who was admitted into the hospital within the past 3 mo receives 5 points (including 2 points for being older than 35 y and 1 point for male sex). If this person also has rheumatoid arthritis (1 point) and a plaster cast of the lower leg (4 points), this results in a total of 10 points. In our plaster cast population, the score ranged between 4 and 20 points (out of a maximum of 29 points for men and 35 points for women). In all, 59.6% (*n =* 137) of the plaster cast patients had a score of at least 10 points. Fig 3 shows the distribution of individual L-TRiP(cast) scores among cases and controls.

**Table 4:** L-TRiP(cast) score based on the clinical risk prediction model.

Environmental Predictor Variable	Point Value
Age ≥ 35 and < 55 y	2
Age ≥ 55 y	3
Male sex	1
Current use of oral contraceptives	4
Cancer within the past 5 y	3
Pregnancy or puerperium	3
BMI ≥ 25 and < 35 kg/m^2^	1
BMI ≥ 35 kg/m^2^	2
Pneumonia	3
Family history of VTE (first-degree relative)	2
Comorbidity (rheumatoid arthritis, chronic kidney disease, COPD, multiple sclerosis)	1
Hospital admission within the past 3 mo	2
Bedridden within the past 3 mo	2
Surgery within the past 3 mo	2
Superficial vein thrombosis	3
Plaster cast: complete leg	5
Plaster cast: circular knee cast (ankle free)	2
Plaster cast: foot	2
Plaster cast: lower leg	4

This L-TRiP(cast) score was derived from the regression coefficients (betas) of the clinical prediction model: 0.20 < beta ≤ 0.75, 1 point; 0.75 < beta ≤ 1.25, 2 points; 1.25 < beta ≤ 1.75, 3 points; 1.75 < beta ≤ 2.25, 4 points; beta > 2.25, 5 points

In the plaster cast patients, the L-TRiP(cast) score had an AUC of 0.76 (95% CI 0.66–0.86). Using a cutoff point of 10 points (59.6% of patients) to stratify individuals into high versus low risk categories, the sensitivity was 65.1%, and the specificity was 72.2%. Assuming an incidence of VTE of 2.5%, the positive predictive value of the test was 5.7%, and the negative predictive value was 98.8%. Table 5 shows predictive values that were calculated for different cutoff points.

**Table 5:** Predictive performance of the L-TRiP(cast) score in plaster cast patients.

Cutoff Point	Percent Positive	Sensitivity	Specificity	Sensitivity + Specificity	Positive Predictive Value*	Negative Predictive Value*	Likelihood Positive	Likelihood Negative
2	100.0%	100.0%	0.0%	100.0%	2.5%	99.2%	1.0	0.3
3	100.0%	100.0%	0.1%	100.0%	2.5%	99.2%	1.0	0.3
4	99.9%	100.0%	0.1%	100.0%	2.5%	98.6%	1.0	0.5
5	99.3%	99.6%	2.0%	101.6%	2.5%	99.5%	1.0	0.2
6	96.5%	98.4%	14.2%	112.6%	2.9%	99.7%	1.1	0.1
7	92.1%	95.3%	26.2%	121.5%	3.2%	99.5%	1.3	0.2
8	87.8%	92.6%	39.7%	132.2%	3.8%	99.5%	1.5	0.2
9	74.7%	80.8%	60.8%	141.7%	5.0%	99.2%	2.1	0.3
10	59.6%	65.1%	72.2%	137.2%	5.7%	98.8%	2.3	0.5
11	44.4%	49.0%	82.0%	131.0%	6.5%	98.4%	2.7	1.0
12	31.2%	34.5%	88.3%	122.9%	7.1%	98.1%	3.0	0.7
13	21.7%	24.8%	96.3%	121.1%	14.7%	98.0%	6.7	0.8
14	14.3%	16.2%	96.6%	112.8%	10.9%	97.8%	4.7	0.9

*Presuming a prevalence of VTE in plaster cast patients of 2.5%.

### Validation Cohorts

The characteristics of the THE-VTE study population, with 784 cases and 523 controls in our analyses, were similar to those of our derivation cohort. DVT was found in 460 (59%) cases, and PE (with or without DVT) in 325 (41%) cases. Plaster cast of the lower extremity was present in 32 (4.1%) cases and seven (1.3%) controls. In the Milan study, plaster cast of the lower extremity was seen in 143 (8.1%) cases and eight (0.4%) controls.

As discussed above, when selecting predictors for our restricted model, we selected variables based on availability in the validation cohorts without reducing the AUC performance. Because the MILAN study lacked data on Von Willebrand factor levels, monocyte percentage, varicose veins, and hospital admission within the past 3 mo (which were strong predictors in the derivation cohort), we adjusted our restricted model. These predictors were replaced with BMI, prothrombin mutation, non-O blood type, and bedridden within the past 3 mo. The predictive AUC value of this adjusted restricted model performed similarly to the unadjusted model in the MEGA study population. Therefore, we chose to continue using these predictors in our restricted model.

Results of the validation of the different prediction scores can be found in Table 6. The clinical model showed an AUC of 0.75 (95% CI 0.55–0.94) in plaster cast patients in the THE-VTE cohort. In the Milan study population, AUCs were 0.93 (95% CI 0.86–1.00), 0.92 (95% CI 0.87–0.98), and 0.96 (95% CI 0.92–0.99) for the full, restricted, and clinical models, respectively, in plaster cast patients. The L-TRiP(cast) score performed very well, with AUCs of 0.95 (95% CI 0.91–0.99) and 0.77 (95% CI 0.58–0.96) in the Milan study and the THE-VTE study, respectively.

**Table 6:** Validation results in plaster cast patients.

Model or Prediction Score	AUC (95% CI)	
	THE-VTE Study	Milan Study
Full model	—	0.93 (0.86–1.00)
Restricted model	—	0.92 (0.87–0.98)
Clinical model	0.75 (0.55–0.94)	0.96 (0.92–0.99)
L-TRiP(cast) score	0.77 (0.58–0.96)	0.95 (0.91–0.99)

## Discussion

### Summary of Key Findings

In this study we developed a prediction model for the occurrence of VTE in patients with plaster cast immobilization of the lower extremity. Due to the wide range of incidence rates that have been reported and a considerable bleeding risk secondary to anticoagulant prophylaxis, current guidelines on thromboprophylaxis are contradictory. A prediction model could help clinicians decide whether or not to prescribe thromboprophylaxis in individual patients [24,25].

The full model performed best in our derivation cohort, with an AUC of 0.85 (95% CI 0.77–0.92), and consisted of a mix of environmental risk factors, genetic risk factors, and biomarkers. However, as measurement of biomarkers and SNPs can be difficult, expensive, or take some time in clinical practice, we also developed two reduced versions of this full model: a restricted model and a clinical model. These models are more practical for clinical use and still showed good predictive characteristics, with an AUC of 0.84 (95% CI 0.77–0.92) and 0.77 (95% CI 0.66–0.87) for the restricted model (only one biomarker and two SNPs included) and the clinical model (no biomarkers or SNPs), respectively. In validation studies, the clinical and restricted models performed well in two validation populations. Of all the models, the clinical model performed best, with an AUC of 0.75 (95% CI 0.55–0.94) and 0.96 (95% CI 0.92–0.99) in the THE-VTE study and the Milan study, respectively.

### Previous Prediction Models

Whereas other studies have examined risk factors and developed prediction models for thrombosis in the general population, this study focused particularly on the development of VTE in plaster cast patients. Considering the low risk of a first event and the heterogeneous etiology of VTE, it is not efficient to develop a prediction model for the general population. Instead, targeting a specific high risk group is much more likely to lead to a model that can be used in clinical practice to distinguish individuals in whom the expected risk is sufficiently high to warrant thromboprophylactic therapy [1]. For instance, location of the plaster cast (complete leg, lower leg, etc.) was the most important predictive variable in our target group, giving specific information for these patients.

The predictive value of genetic and environmental risk factors for VTE has been described in previous studies [3,4,26]. Hippisley-Cox and Coupland reported an increased risk of VTE in the general population in association with overweight, COPD, varicose veins, congestive heart failure, chronic renal disease, cancer, inflammatory bowel disease, hospital admission within the past 6 mo, use of antipsychotic drugs, use of oral contraceptives, use of hormone replacement therapy, use of tamoxifen, and smoking, which resulted in an AUC value of 0.75 (95% CI 0.74–0.76) in their validation cohort, which is in line with our results [4]. However, one very well established risk factor, i.e., immobilization, was not incorporated into this model. de Haan et al. recently found that multiple SNP testing had an additional predictive value in the prediction of VTE compared with a model with environmental variables only (also partially MEGA study data) [6]. They identified five common SNPs and incorporated these variables into a prediction model for the general population, together with environmental risk factors. This model had an AUC of 0.77 (95% CI 0.74–0.80) [6].

There have been only a few studies, predominantly in cancer-induced thrombosis, that have investigated the predictive role of biomarkers, such as high factor VIII and prothrombin fragment 1 + 2, in the prediction of VTE [7,9]. While other studies have focused on environmental risk factors, genetic risk factors, or biomarkers only, we incorporated all three types of predictor variables into our model. So far, this is the only prediction model for VTE to our knowledge that has combined all of these variables and that has focused on plaster cast patients.

### Limitations of the Study

Although we incorporated genetic risk factors, environmental risk factors, and biomarkers in our model, we were not able to include age and sex as predictor variables at first, since the controls in our study were frequency matched on age and sex. To overcome this, control individuals were weighted to the age and sex distribution of the Dutch population, which made it possible to estimate the real effect of age and sex on the risk of VTE in our case–control study. We performed a sensitivity analysis with and without weighting of control individuals: the results for the weighted analyses were equal to those of the unweighted analyses in both the derivation and validation studies. This way, age and sex were incorporated into our models as predictor variables, making our risk score suitable for patients from 18 up to 70 y old. Another limitation of the study was that blood collection was performed after the occurrence of thrombosis. As a result, the levels of coagulation factors may have been a consequence of the thrombosis rather than a cause. However, increased levels of factor VIII and fibrinogen measured after the occurrence of thrombosis have been shown not to be due to acute phase reactions [27]. In fact, high factor VIII levels seem to be a permanent phenomenon, and repeated measurements of factor VIII show little variation [28,29]. A third limitation was that general information on anticoagulation therapy was available, but information on possible thromboprophylaxis during plaster cast was missing. Nonetheless, if we look at the results of a survey on thromboprophylaxis conducted in the Netherlands in 2002, which overlaps with the inclusion period of our study, 30% of orthopedic surgeons provided thromboprophylaxis during lower leg plaster cast, and 88% during complete leg plaster cast [30]. Therefore, VTE risk may have been underestimated in this study. A fourth limitation of the study is that the relatively small number of individuals with plaster cast (*n =* 230) hinders development of a prediction model specifically targeted to this group. To overcome this issue and avoid overfitting, we first developed our model in the entire MEGA study population and then tested our full model in the plaster cast subgroup. Finally, using a c-statistic alone for building a prediction model may eliminate important risk factors. To overcome this, we first developed our full model based on clinical as well as statistical criteria. Candidate predictors were retained based on (1) a forward selection procedure or (2) well-established association in the literature. We used the c-statistic only to slim down our full model so that the same predictive power could be reached with fewer predictor variables.

### Clinical Implications

Our study showed a good performance of the different prediction models in plaster cast patients. Although we found an added value of genetic variance and biomarker information in the prediction of VTE, the clinical model (with environmental factors only) performed only slightly less well than the full model, with a good discriminative statistic of 0.77 (95% CI 0.66–0.87) in the derivation data. Moreover, in our validation sets, the clinical model performed as well or even better than the full model, with an AUC of 0.75 (95% CI 0.55–0.94) and 0.96 (95% CI 0.92–0.99) in the THE-VTE study and the Milan study, respectively. Therefore, it is doubtful whether information on genetic variance and biomarkers will lead to higher accuracy in the prediction algorithm. In addition, genetic testing is currently not practical in the clinical setting and probably less cost-effective (due to the small prevalence of some genetic variants), and therefore the diagnostic value of these predictors might be limited.

Currently, the American College of Chest Physicians advises that pharmacologic thromboprophylaxis should not be used in patients with isolated lower leg injuries requiring leg immobilization [12]. The UK National Institute for Health Care and Excellence guidelines recommend considering VTE prophylaxis after evaluating the risks and benefits in clinical discussion with the patient [31]. In addition, the British Society for Haematology recommends prophylaxis for patients at high risk of VTE associated with lower limb plaster cast [32]. Our L-TRiP(cast) score, based on the clinical model, classifies individuals with plaster cast of the lower extremity as high risk or low risk for VTE. This may give guidance to clinicians on prescribing thromboprophylaxis, in line with the latest guidelines. Defining a definite cutoff point is not straightforward. We cautiously suggest using a cutoff point of 9 points to classify individuals as being at high risk for VTE, in which case 74.7% of the people with plaster cast (cases and controls) in our study were identified as high risk. In this way, our risk score can identify a large proportion of people at risk; assuming an overall incidence of VTE of 2.5% (or more with increasing age), the model in these patients has a positive predictive value for the development of VTE of 5.0% while only 0.8% of individuals who scored lower than 9 points will develop VTE. For recurrence, a ≥5.0% risk is considered as an indication for thromboprophylaxis [33], which outweighs the risk of major bleeding. For short term treatment (~6 wk for plaster cast), the bleeding risk is obviously much lower and is estimated at 0.5%. Furthermore, a higher sensitivity could be preferred over a higher specificity, as the burden of missing a VTE might be worse than the burden of overtreatment (i.e., prophylaxis without therapeutic consequences and bleeding complications). While an established cutoff is lacking, clinicians may determine the trade-off between thrombosis and bleeding risk using this decision rule, until additional results from other studies are available (ideally, a randomized controlled trial that compares thromboprophylaxis in all plaster cast patients, or never thromboprophylaxis, with the decision rule based on our L-TRiP[cast] score).

### Conclusion

By using information on environmental risk factors, genetic risk factors, and biomarkers, we were able to develop models that predict the risk of VTE after cast immobilization of the lower extremity. The derivation models in this study show that determination of biomarkers and genetic variance leads to better accuracy in the prediction of VTE in plaster cast patients. However, the validation data show that the clinical model performs as well, or even better. The L-TRiP(cast) score may therefore be more efficient and can be used in the clinical setting. These results can give guidance in clinical decision-making until an unambiguous guideline for thromboprophylaxis therapy in these patients is available, so that not every patient needs to be exposed to the risk and burden of anticoagulant treatment.

## Supporting Information

S1 Analysis Plan Analysis plan for the development of the L-TRiP(cast) score. (DOCX)

S1 Data Overview of missing data and multiple imputation. (DOCX)

S1 Laboratory Analyses Detailed information on laboratory analyses. (DOCX)

S1 Table Basic characteristics of cases and controls of the derivation cohort. (XLSX)

S2 Table Univariate and multivariate analyses showing ORs for VTE, comparing cases and controls. (XLSX)

S1 Weights Table of weights assigned to different strata for control individuals. (XLSX)

## Abbreviations

AUC: area under the curve
BMI: body mass index
COPD: chronic obstructive pulmonary disease
DVT: deep vein thrombosis
OR: odds ratio
PE: pulmonary embolism
ROC: receiver operating characteristic
SNP: single nucleotide polymorphism
TIA: transient ischemic attack
VTE: venous thromboembolism
