# Supplementary material for: Venous Thrombosis Risk after Cast Immobilization of the Lower Extremity: Derivation and Validation of a Clinical Prediction Score, L-TRiP(cast), in Three Population-Based Case–Control Studies
Source: PLoS Med. 2015 Nov 10;12(11):e1001899. doi: 10.1371/journal.pmed.1001899 (PMC4640574; doi:10.1371/journal.pmed.1001899)
Supplement: S1 Analysis Plan — (DOCX) [file pmed.1001899.s001.docx]

**S1 Analysis Plan. Analysis plan for the development of the L-TriP(cast) score**

The MEGA study is a case-control study, set up to assess the risk of venous thrombosis for several risk factors (including interaction between risk factors). Common (acquired) risk factors, biomarkers and genetic risk factors were measured through questionnaires and blood sampling. Causal relationships were estimated by calculating relative risks (by means of the odds ratio). Cases were identified from six anticoagulation clinics in the Netherlands and initially control subjects were partners of cases. Because not all cases had a partner or some partners refused to cooperate, it was decided to include extra controls, who were identified using a random digit dialing method.

In the past years many risk factors have been identified using the MEGA data (see publication list below [1–51]). This information and that of other studies has led to current knowledge on dozens of risk factors for venous thrombosis. Combining these risk factors now allows identifying patients at high risk of developing venous thrombosis, in certain situations. Hence, in recent years, the focus of the MEGA-study has shifted from identifying separate risk factors towards prediction of thrombosis, i.e. identification of high-risk groups. This development is a logical and direct result of the knowledge that was gained during the past years.

The main goal of the current project was to develop a prediction model for venous thrombosis for patients with plaster cast of the lower extremity. The MEGA database was the ideal source of information for this study (case-control database with all information on biomarkers, acquired and genetic risk factors for venous thrombosis). As planned we developed a multivariate logistic regression model to identify predictors. Risk factor selection was based both on clinical importance and the strength of the causal relationship. (This analysis has been described in detail in the manuscript itself). A validation study performed in two databases was added in a later stage to demonstrate its external validity for the general population (and to show that results were not just data-driven). During the review process it was decided to weigh all controls subjects to the age and sex distribution of the Netherlands in 2001, which was necessary due to the age- and (opposite) sex-matching. This way, age and sex could be incorporated as predictors in de model (which improved the clinical usefulness of the L-TRiP(cast) score).

Reference List

1. Flinterman LE, van Hylckama Vlieg A, Rosendaal FR, Cannegieter SC. Body height, mobility, and risk of first and recurrent venous thrombosis. J Thromb Haemost. 2015;13: 548-554.

2. Timp JF, Lijfering WM, Flinterman LE, van Hylckama Vlieg A, Le Cessie S, Rosendaal FR et al. Predictive value of factor VIII levels for recurrent venous thrombosis: results from the MEGA follow-up study. J Thromb Haemost. 2015.

3. van Adrichem RA, Nelissen RG, Schipper IB, Rosendaal FR, Cannegieter SC. Risk of venous thrombosis after arthroscopy of the knee: results from a large population-based case-control study. J Thromb Haemost. 2015;13: 1441-1448.

4. Vuckovic BA, van RN, Cannegieter SC, Rosendaal FR, Lijfering WM. Vitamin supplementation on the risk of venous thrombosis: results from the MEGA case-control study. Am J Clin Nutr. 2015;101: 606-612.

5. Yap ES, Timp JF, Flinterman LE, van Hylckama Vlieg A, Rosendaal FR, Cannegieter SC et al. Elevated levels of factor VIII and subsequent risk of all-cause mortality: results from the MEGA follow-up study. J Thromb Haemost. 2015.

6. de Haan HG, Bezemer ID, Vossen CY, van Hylckama Vlieg A, Boehringer S, Hasstedt SJ et al. Genetic variants in Cell Adhesion Molecule 1 (CADM1): a validation study of a novel endothelial cell venous thrombosis risk factor. Thromb Res. 2014;134: 1186-1192.

7. Debeij J, van ZB, Dekkers OM, Doggen CJ, Smit JW, van Zanten AP et al. High levels of procoagulant factors mediate the association between free thyroxine and the risk of venous thrombosis: the MEGA study. J Thromb Haemost. 2014;12: 839-846.

8. Rezende SM, Lijfering WM, Rosendaal FR, Cannegieter SC. Hematologic variables and venous thrombosis: red cell distribution width and blood monocyte count are associated with an increased risk. Haematologica. 2014;99: 194-200.

9. Roach RE, Lijfering WM, Rosendaal FR, Cannegieter SC, Le Cessie S. Sex difference in risk of second but not of first venous thrombosis: paradox explained. Circulation. 2014;129: 51-56.

10. van Adrichem RA, Debeij J, Nelissen RG, Schipper IB, Rosendaal FR, Cannegieter SC. Below-knee cast immobilization and the risk of venous thrombosis: results from a large population-based case-control study. J Thromb Haemost. 2014;12: 1461-1469.

11. van Hylckama Vlieg A, Flinterman LE, Bare LA, Cannegieter SC, Reitsma PH, Arellano AR et al. Genetic variations associated with recurrent venous thrombosis. Circ Cardiovasc Genet. 2014;7: 806-813.

12. Ocak G, Vossen CY, Verduijn M, Dekker FW, Rosendaal FR, Cannegieter SC et al. Risk of venous thrombosis in patients with major illnesses: results from the MEGA study. J Thromb Haemost. 2013;11: 116-123.

13. Ocak G, Lijfering WM, Verduijn M, Dekker FW, Rosendaal FR, Cannegieter SC et al. Risk of venous thrombosis in patients with chronic kidney disease: identification of high-risk groups. J Thromb Haemost. 2013;11: 627-633.

14. Pintao MC, Ribeiro DD, Bezemer ID, Garcia AA, de Visser MC, Doggen CJ et al. Protein S levels and the risk of venous thrombosis: results from the MEGA case-control study. Blood. 2013;122: 3210-3219.

15. Roach RE, Lijfering WM, Flinterman LE, Rosendaal FR, Cannegieter SC. Increased risk of CVD after VT is determined by common etiologic factors. Blood. 2013;121: 4948-4954.

16. Roach RE, Lijfering WM, Helmerhorst FM, Cannegieter SC, Rosendaal FR, van Hylckama Vlieg A. The risk of venous thrombosis in women over 50 years old using oral contraception or postmenopausal hormone therapy. J Thromb Haemost. 2013;11: 124-131.

17. de Haan HG, Bezemer ID, Doggen CJ, Le Cessie S, Reitsma PH, Arellano AR et al. Multiple SNP testing improves risk prediction of first venous thrombosis. Blood. 2012;120: 656-663.

18. Flinterman LE, van Hylckama Vlieg A, Cannegieter SC, Rosendaal FR. Long-term survival in a large cohort of patients with venous thrombosis: incidence and predictors. PLoS Med. 2012;9: e1001155.

19. Ribeiro DD, Lijfering WM, van Hylckama Vlieg A, Rosendaal FR, Cannegieter SC. Pneumonia and risk of venous thrombosis: results from the MEGA study. J Thromb Haemost. 2012;10: 1179-1182.

20. Roach RE, Siegerink B, Le Cessie S, Rosendaal FR, Cannegieter SC, Lijfering WM. Coffee consumption is associated with a reduced risk of venous thrombosis that is mediated through hemostatic factor levels. J Thromb Haemost. 2012;10: 2519-2525.

21. Stegeman BH, Helmerhorst FM, Vos HL, Rosendaal FR, van Hylckama Vlieg A. Sex hormone-binding globulin levels are not causally related to venous thrombosis risk in women not using hormonal contraceptives. J Thromb Haemost. 2012;10: 2061-2067.

22. Stuijver DJ, Debeij J, van ZB, Dekkers OM, Smit JW, Buller HR et al. Levels of prolactin in relation to coagulation factors and risk of venous thrombosis. Results of a large population-based case-control study (MEGA-study). Thromb Haemost. 2012;108: 499-507.

23. van Langevelde K, Flinterman LE, van Hylckama Vlieg A, Rosendaal FR, Cannegieter SC. Broadening the factor V Leiden paradox: pulmonary embolism and deep-vein thrombosis as 2 sides of the spectrum. Blood. 2012;120: 933-946.

24. van Langevelde K, Lijfering WM, Rosendaal FR, Cannegieter SC. Increased risk of venous thrombosis in persons with clinically diagnosed superficial vein thrombosis: results from the MEGA study. Blood. 2011;118: 4239-4241.

25. Arellano AR, Bezemer ID, Tong CH, Catanese JJ, Devlin JJ, Reitsma PH et al. Gene variants associated with venous thrombosis: confirmation in the MEGA study. J Thromb Haemost. 2010;8: 1132-1134.

26. Meltzer ME, Lisman T, de Groot PG, Meijers JC, Le Cessie S, Doggen CJ et al. Venous thrombosis risk associated with plasma hypofibrinolysis is explained by elevated plasma levels of TAFI and PAI-1. Blood. 2010;116: 113-121.

27. Bezemer ID, van der Meer FJ, Eikenboom JC, Rosendaal FR, Doggen CJ. The value of family history as a risk indicator for venous thrombosis. Arch Intern Med. 2009;169: 610-615.

28. Bezemer ID, Arellano AR, Tong CH, Rowland CM, Ireland HA, Bauer KA et al. F9 Malmo, factor IX and deep vein thrombosis. Haematologica. 2009;94: 693-699.

29. Kuipers S, Cannegieter SC, Doggen CJ, Rosendaal FR. Effect of elevated levels of coagulation factors on the risk of venous thrombosis in long-distance travelers. Blood. 2009;113: 2064-2069.

30. Li Y, Bezemer ID, Rowland CM, Tong CH, Arellano AR, Catanese JJ et al. Genetic variants associated with deep vein thrombosis: the F11 locus. J Thromb Haemost. 2009;7: 1802-1808.

31. Pomp ER, Doggen CJ, Vos HL, Reitsma PH, Rosendaal FR. Polymorphisms in the protein C gene as risk factor for venous thrombosis. Thromb Haemost. 2009;101: 62-67.

32. Ramcharan AS, Van Stralen KJ, Snoep JD, Mantel-Teeuwisse AK, Rosendaal FR, Doggen CJ. HMG-CoA reductase inhibitors, other lipid-lowering medication, antiplatelet therapy, and the risk of venous thrombosis. J Thromb Haemost. 2009;7: 514-520.

33. Schreijer AJ, Cannegieter SC, Doggen CJ, Rosendaal FR. The effect of flight-related behaviour on the risk of venous thrombosis after air travel. Br J Haematol. 2009;144: 425-429.

34. van Hylckama Vlieg A, Helmerhorst FM, Vandenbroucke JP, Doggen CJ, Rosendaal FR. The venous thrombotic risk of oral contraceptives, effects of oestrogen dose and progestogen type: results of the MEGA case-control study. BMJ. 2009;339: b2921.

35. Bezemer ID, Bare LA, Doggen CJ, Arellano AR, Tong C, Rowland CM et al. Gene variants associated with deep vein thrombosis. JAMA. 2008;299: 1306-1314.

36. Coppens M, Reijnders JH, Middeldorp S, Doggen CJ, Rosendaal FR. Testing for inherited thrombophilia does not reduce the recurrence of venous thrombosis. J Thromb Haemost. 2008;6: 1474-1477.

37. Le Cessie S, Nagelkerke N, Rosendaal FR, Van Stralen KJ, Pomp ER, van Houwelingen HC. Combining matched and unmatched control groups in case-control studies. Am J Epidemiol. 2008;168: 1204-1210.

38. Meltzer ME, Lisman T, Doggen CJ, de Groot PG, Rosendaal FR. Synergistic effects of hypofibrinolysis and genetic and acquired risk factors on the risk of a first venous thrombosis. PLoS Med. 2008;5: e97.

39. Pomp ER, Rosendaal FR, Doggen CJ. Alcohol consumption is associated with a decreased risk of venous thrombosis. Thromb Haemost. 2008;99: 59-63.

40. Pomp ER, Rosendaal FR, Doggen CJ. Smoking increases the risk of venous thrombosis and acts synergistically with oral contraceptive use. Am J Hematol. 2008;83: 97-102.

41. Pomp ER, Lenselink AM, Rosendaal FR, Doggen CJ. Pregnancy, the postpartum period and prothrombotic defects: risk of venous thrombosis in the MEGA study. J Thromb Haemost. 2008;6: 632-637.

42. Tick LW, Kramer MH, Rosendaal FR, Faber WR, Doggen CJ. Risk factors for post-thrombotic syndrome in patients with a first deep venous thrombosis. J Thromb Haemost. 2008;6: 2075-2081.

43. Van Stralen KJ, Rosendaal FR, Doggen CJ. Minor injuries as a risk factor for venous thrombosis. Arch Intern Med. 2008;168: 21-26.

44. Van Stralen KJ, Doggen CJ, Bezemer ID, Pomp ER, Lisman T, Rosendaal FR. Mechanisms of the factor V Leiden paradox. Arterioscler Thromb Vasc Biol. 2008;28: 1872-1877.

45. Bezemer ID, Doggen CJ, Vos HL, Rosendaal FR. No association between the common MTHFR 677C->T polymorphism and venous thrombosis: results from the MEGA study. Arch Intern Med. 2007;167: 497-501.

46. Pomp ER, Le Cessie S, Rosendaal FR, Doggen CJ. Risk of venous thrombosis: obesity and its joint effect with oral contraceptive use and prothrombotic mutations. Br J Haematol. 2007;139: 289-296.

47. Rosendaal FR, van Hylckama Vlieg A, Doggen CJ. Venous thrombosis in the elderly. J Thromb Haemost. 2007;5 Suppl 1: 310-317.

48. Van Stralen KJ, Le Cessie S, Rosendaal FR, Doggen CJ. Regular sports activities decrease the risk of venous thrombosis. J Thromb Haemost. 2007;5: 2186-2192.

49. Cannegieter SC, Doggen CJ, van Houwelingen HC, Rosendaal FR. Travel-related venous thrombosis: results from a large population-based case control study (MEGA study). PLoS Med. 2006;3: e307.

50. Chinthammitr Y, Vos HL, Rosendaal FR, Doggen CJ. The association of prothrombin A19911G polymorphism with plasma prothrombin activity and venous thrombosis: results of the MEGA study, a large population-based case-control study. J Thromb Haemost. 2006;4: 2587-2592.

51. Blom JW, Doggen CJ, Osanto S, Rosendaal FR. Malignancies, prothrombotic mutations, and the risk of venous thrombosis. JAMA. 2005;293: 715-722.
