# Supplementary material for: Venous Thrombosis Risk after Cast Immobilization of the Lower Extremity: Derivation and Validation of a Clinical Prediction Score, L-TRiP(cast), in Three Population-Based Case–Control Studies
Source: PLoS Med. 2015 Nov 10;12(11):e1001899. doi: 10.1371/journal.pmed.1001899 (PMC4640574; doi:10.1371/journal.pmed.1001899)
Supplement: S1 Data — (DOCX) [file pmed.1001899.s002.docx]

**S1 Data. Overview of missing data and multiple imputation.**

Multiple imputation was used to complete missing predictor values, of which the list below gives an overview. Data on environmental risk factors was collected by means of a questionnaire, missing data on the questionnaire resulted in this missing data. Blood collection was terminated for logistic reasons on May 31, 2002. For participants included after this date no blood was sampled which resulted in missing data which is quite likely completely at random. For patients included after May 31, 2002, buccal swabs were collected for DNA analyses. Patients who did not return their buccal swab had missing data for the DNA variables.

|  | **Percentage missing %** |
| --- | --- |
| **Environmental predictor variables** |  |
| Smoking (current) | **6.2** |
| Varicose veins | **15.5** |
| Cancer within the past 5 years | **0** |
| Congestive heart failure | **9.1** |
| Body Mass Index BMI>25 | **7.6** |
| Cerebrovascular events | **9.0** |
| Family history of VTE | **27.8** |
| Hospital admission within the past 3 months | **0** |
| Bedridden within the past 3 months | **0.8** |
| Paralysis (partial) | **9.1** |
| Surgery within the past 3 months | **0.5** |
| Pregnancy or puerperium | **2.3** |
| Current use of antipsychotic medication | **0** |
| Current use of tamoxifen | **0** |
| Current use of hormonal replacement therapy | **1.8** |
| Current use of oral contraceptives | **0.5** |
| Superficial vein thrombosis | **9.0** |
| Plaster cast | **0** |
|  |  |
| **Hemorheologic and coagulation predictor variables** |  |
| Factor VIII activity | **54.0** |
| Von Willebrand Factor | **54.0** |
| Factor XI activity | **54.0** |
| Percentage monocytes | **54.8** |
| Red cell Distribution With (RDW) | **54.5** |
| Total cysteine | **54.2** |
|  |  |
|  |  |
| **Genetic predictor variables** |  |
| Factor V Leiden mutation | **18.7** |
| Prothrombin mutation | **18.6** |
| ABO Bloodtype | **25.1** |
