# Supplementary material for: Venous Thrombosis Risk after Cast Immobilization of the Lower Extremity: Derivation and Validation of a Clinical Prediction Score, L-TRiP(cast), in Three Population-Based Case–Control Studies
Source: PLoS Med. 2015 Nov 10;12(11):e1001899. doi: 10.1371/journal.pmed.1001899 (PMC4640574; doi:10.1371/journal.pmed.1001899)
Supplement: S1 Laboratory Analyses — (DOCX) [file pmed.1001899.s003.docx]

**S1 Laboratory Analyses. Detailed information on laboratory analyses.**

Coagulation markers such as prothrombin (factor II [FII]) activity, factor VII (FVII) activity, factor VIII (FVIII) activity, antithrombin (AT) activity, protein C (PC) activity and protein S (PS) antigen level were measured with a mechanical clot detection method on a STA-R coagulation analyzer following the instructions of the manufacturer (Diagnostica Stago, Asnieres, France). Levels of factor IX antigen (FIX) were determined by enzyme-linked immunosorbent assay (ELISA). Fibrinogen activity was measured on the STA-R analyzer according to methods of Clauss. In the presence of excess thrombin, the coagulation time of a diluted plasma sample was measured. von Willebrand factor (VWF) antigen was measured with the immunoturbidimetric method, using the STA Liatest kit (rabbit anti–human VWF antibodies), following the instructions of the manufacturer (Diagnostica Stago). Hemorheologic markers such as hematocrit, white blood cell count (WBCC), percentage/number lymphocytes, percentages/number monocytes, percentage/number granulocytes, red blood cell count (RBCC), hemoglobin level, mean cell volume (MCV), mean cell hemoglobin (MCH), mean cell hemoglobin concentration (MCHC), red cell distribution width (RDW), total homocysteine, total cysteine, methionine and factor X antigen level were measured using the Beckman coulter analyzer.
